# Effects of Lamiaceae family plants and their bioactive ingredients on coronavirus‐induced lung inflammation

**DOI:** 10.1002/fsn3.3903

**Published:** 2023-12-27

**Authors:** Majid Kianmehr, Mohammad Reza Khazdair, Abbasali Abbasnezhad, Muhammad Akram

**Affiliations:** ^1^ Esfarayen Faculty of Medical Sciences Esfarayen Iran; ^2^ Cardiovascular Diseases Research Center Birjand University of Medical Sciences Birjand Iran; ^3^ Applied Biomedical Research Center Mashhad University of Medical Sciences Mashhad Iran; ^4^ Department of Physiology, Faculty of Medicine Gonabad University of Medical Sciences Gonabad Iran; ^5^ Department of Eastern Medicine Government College University Faisalabad Faisalabad Pakistan

**Keywords:** anti‐inflammatory effects, antiviral effects, coronaviruses, medicinal herbs

## Abstract

Coronaviruses (CoVs) are a family of viruses that cause infection in respiratory and intestinal systems. Different types of CoVs, those responsible for the SARS‐CoV and the new global pandemic of coronavirus disease 2019 in people, have been found. Some plants were used as food additives: spices and dietary and/or medicinal purposes in folk medicine. We aimed to provide evidence about possible effects of two Lamiaceae family plants on control or treatment of CoVs‐induced inflammation. The keywords including coronaviruses, *Thymus vulgaris*, *Zataria multiflora*, thymol, carvacrol, antivirus, and anti‐inflammatory and antioxidant effects were searched in various databases such as PubMed, Web of Sciences (ISI), and Google Scholar until September 2022. The medicinal herbs and their main ingredients, thymol and carvacrol, showed antiviral properties and reduced inflammatory mediators, including IL‐1β; IL‐6, and TNF‐α, at both gene and protein levels but increased the levels of IFN‐γ in the serum as anti‐inflammatory cytokine. These medicinal herbs and their constituents also reduce oxidative stress and enhance antioxidant capacity. The results of molecular docking analyses also indicated that polyphenol components such as thymol, carvone, and carvacrol could inhibit the entry of the viruses into the host cells in molecular docking analyses. The antiviral, anti‐inflammatory, and antioxidant effects of these plants may be due to actions of their phenolic compounds that modulate immune response and may be useful in the control and treatment of CoV‐induced lung disorder.

## Graphical abstract

The Lamiaceae family plants were used traditionally for food additives, spices, and dietary and/or medicinal purposes. We provide evidence about possible therapeutic effects of two Lamiaceae family plants on control or treatment of CoVs‐induced inflammation.

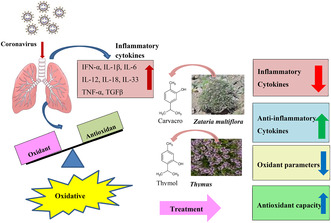

## 1 INTRODUCTION

The viruses from coronaviruses (CoVs) family usually cause infection in respiratory and intestinal systems in animals and humans (Andersen et al., 2020). CoVs usually cause mild colds in people, but they cased, the severe acute respiratory syndrome (SARS) in 2002‐2003; the Middle‐East respiratory syndrome (MERS) in 2012; and at the end of 2019 caused SARS‐CoV‐2 (Malik et al., 2020). Novel coronavirus causes severe respiratory problems and infection in patients (Shanmugaraj et al., 2020). SARS‐CoV‐2 has high transmissibility and infectivity compared to SARS and MERS, despite a low mortality rate (Liu et al., 2020). It has been reported that some herbal products showed anti‐inflammatory and immune‐regulatory effects in folk medicine. So, the isolation of plants' bioactive components with various pharmacological effects occurred in the 19th century (Phillipson, 2001).

Almost 64% of population in the world use medicinal herbs for treatment of various diseases. In addition, almost 50% of synthetic drugs are derived from herbal sources (Newman & Cragg, 2016). The phytochemicals as anti‐infective agents including flavonoids and hydroxylated phenols are effective against infections and could help to defense system to combat pathogens (Dixon et al., 1983). Moreover, alkaloids and flavonoids showed antimicrobial and larvicidal effects (Naghibi et al., 2005). The possible advantageous effects of *Nigella sativa* and its ingredients, thymoquinone, as well as natural products and flavonoids on SARS‐CoV‐2, were also reported (Khazdair, 2021; Khazdair, Anaeigoudari, et al., 2021).

The plants from Lamiaceae family are well known for the presence of diterpenoids and essential oils in many members of the plant family. Some species of this plant family such as *Thymus vulgaris* (TV) and *Zataria multiflora* Boiss (ZM) are used in culinary as well as used as spice, food additive, and flavorings (Naghibi et al., 2005). TV and ZM have been used to treat coughs due to cold, laryngitis, and bronchitis and are also used for the treatment of oral cavity disorders in Iranian folk medicine (Mozaffarian, 1996).

*T. vulgaris* consists of various chemical compounds including phenolic compounds (thiophenol, quininic acid, p‐coumaric acid, caffeic acid, and geranic acid), terpenoids (thymol, carvacrol, linalool, and p‐cymene), flavonoids (luteolin, 6‐hydroxyluteolin, and apigenin), steroids, tannins, alkaloids, and saponins (Patil et al., 2021).

TV is used as herbal medicine to treat inflammation‐induced pain such as insect bites, muscle swelling, and rheumatism by most people, especially in rural areas (Namsa et al., 2009). The antioxidant and anticancer activities of TV extract in the human breast cancer T47D cell line were shown in Heidari et al. (2018). Intraperitoneal administration of TV extract (50, 100, and 200 mg/kg) on bleomycin‐induced pulmonary fibrosis in Wistar rats significantly improved the level of oxidative markers without renal or hepatic cytotoxic effects (Bahri et al., 2022). The constituents of *Z. multiflora* collected from Tehran, Iran, were thymol, carvacrol, p‐cymene, λ‐terpinene, and β‐caryophyllene (Ebrahimzadeh et al., 2003). The main components of *Z. multiflora* obtained from Quetta, Pakistan, were carvacrol, carvacrol acetate, methyl carvacrol, γ‐terpinene, p‐cymene, linalool, and β‐caryophyllene (Ahmad et al., 1999).

ZM is used for analgesic, antiseptic, anthelmintic, and antidiarrheal effects in folk medicine (Ghasemi Dehkordi et al., 2002). ZM has also been used for treatment of dyspepsia vomiting, headache, premature labor pain, and common cold (Amin, 1991). The essential oils of ZM also revealed inhibitory effect on growth of yeasts and showed antimicrobial activities against gram‐positive and ‐negative bacteria (Zomorodian et al., 2011).

ZM and TV have similar chemical and pharmacological properties and are well known as medicinal plants (Amin, 1991). The aims of this review article are to explain the traditional and new pharmacological properties of some medicinal herbs on the respiratory system focusing on molecular immunomodulatory effects, which suggest the possible therapeutic effects on CoVs and suggest a reference for drug development of coronavirus infection.

## 2 METHODS

The data of the current review study were obtained by searching for the terms: coronavirus, *Thymus vulgaris*, *Zataria multiflora*, thymol, carvacrol, antiviral, anti‐inflammatory, and antioxidant effect in the electronic databases, including PubMed, ISI, and Google Scholar until September 2022.

## 3 IMMUNOREGULATORY EFFECTS OF MEDICINAL PLANTS

It has been known that deregulation of immune system is the main cause of many diseases. So, the useful therapeutic approach for the management of various diseases is the management of immune responses (Su et al., 2012). Some natural products and medicinal herbs can affect the function of the immune system by modulation of immune cell activities as well as production of mediators (Das et al., 2004). The quantitative comparison of immunomodulatory properties with respect to T helper cells (Th1) and Th2 of saffron and black seed, as well as their main constituent, were reported (Khazdair, Gholamnezhad, et al., 2021).

## 4 PHARMACOLOGICAL EFFECTS OF TV

TV *is a perennial* flowering plant that widely grows in western Mediterranean and southern Europe (Letchamo & Gosselin, 1996). The plant has been used as a culinary herb, with antiseptic, analgesic, antimicrobial, and antioxidative properties (Baranauskiene et al., 2003). TV is used to treat diabetes, digestive upset, and cough due to cold and chest infections (Ekoh et al., 2014). The oils from TV contain terpenoid, carvacrol, and thymol as phenol compound that has antiviral, antibacterial, antispasmodic, and antioxidative effects (Höferl et al., 2009). *Thymus* spp. also are used for their antiviral, antifungal, antimicrobial, antispasmodic, and diaphoretic effects in traditional medicine (Fachini‐Queiroz et al., 2012). The antibacterial effects of the TV oils against the gram‐negative and gram‐positive bacteria have been reported (Marino et al., 1999).

### 4.1 Antiviral effects of TV

Some plants, their fractions, extracts, derivations, or essential oils, and their possible antiviral prosperities have been reported (Jassim & Naji, 2003). The antiviral effects of thyme spp. including *T. vulgaris*, *T. hyemalis*, and *T. zygis* extracts in contradiction of the herpes simplex virus 1 (HSV‐1) at different stages during virus infection were evaluated. Results showed that pretreated cells with the thyme extract significantly reduced virus infectivity. *T. zygis* extract was also more effective than the other plant extract. The antiviral activity of *Thyme* spp. could be related to the percentage of phenolic compounds (thymol and carvacrol) that are presented in these extracts. These combined extracts mostly inhibited intracellular replication of HSV‐1, although they were able to disrupt the attachment step of the virus (Santoyo et al., 2014). The antiviral efficacy of TV essential oils on feline infectious peritonitis (FIP) (a disease caused by a Coronavirus) showed essential oils of the plant (27 μg/mL) significantly inhibited virus replication. Moreover, the plant essential oils (27 and 270 μg/mL) reduced virucidal activity by reducing viral titer to 1 h of time contact (Catella et al., 2021).

Antiviral activity of monoterpene compounds including α‐terpinene, *p*‐cymen, γ‐terpinen, thymol, and citral against HSV‐1 in vitro was examined. These monoterpenes reduced viral infectivity and inhibited HSV by >80%. In addition, these monoterpenes showed high anti‐HSV‐1 effects by direct inactivation with virus particles. The monoterpenes also interact with herpesvirus particles inactivating viral infection (Astani et al., 2010). The antiviral activity of some monoterpenes such as β‐pinene and limonene against HSV‐1 showed high anti‐HSV‐1 activity and reduced viral infectivity by 100% by interaction with free virus particles (Astani & Schnitzler, 2014). Essential oils from three plants including thyme, camomile, and ginger exhibited antiviral effects against HSV type 2 (HSV‐2) using a plaque reduction on RC‐37 cells. The essential oils at noncytotoxic concentrations significantly reduced plaque formation by about 90%. These results indicated that the obtained essential oils affected HSV‐2 mostly before adsorption maybe by interaction with envelope of the viruses (Koch et al., 2008).

In a study, the antiviral properties of combination of honey and thyme extracts were evaluated. This combination therapy of monkey kidney cell (MKC) culture infected with the rubella virus showed good antirubella activity (Zeina et al., 1996). TV extracts (50 μg/mL) showed antiviral effects in embryonated eggs against Newcastle disease virus (NDV) (Rezatofighi et al., 2014). The results of a cohort study with 161 patients testing positive PCR for COVID‐19 showed that TV (5 g in 100 mL in hot water) every 8 h has a positive effect on cough, anorexia, chest pain, dyspnea, fever, ageusia, and anosmia. There was a significant decrease in blood urea levels and neutrophils. In addition, the lymphocyte count and serum calcium level increased significantly after 3 days of treatment in 86 patients who were exposed compared to 75 patients who were not exposed (Yiagnigni Mfopou et al., 2021).    The effect of TV on the clinical symptoms of COVID‐19 patients (*n* = 83) showed consumption of thyme essential oils (5 mL) three times per day could be useful for reducing fever, cough, headache, dyspnea, weakness, muscular pain, anorexia, fatigue, and chest wall pain. Moreover, blood urea nitrogen, calcium, and neutrophil count were remarkably decreased, while lymphocyte count was increased (Sardari et al., 2021).

The effects of some medicinal herbs' essential oils on the expression of transient receptor potential (TRP) gene family as well as replication of coronavirus were reported (Ulasli et al., 2014). The molecular douching study indicated that interaction between monoterpenoids such as carvone, carvacrol, thymol, and SARS‐CoV‐2 spike protein together with the cellular proteases TMPRSS2, cathepsin L (CatL), and cathepsin B (CatB) showed that the mentioned compounds have most effective molecule against all targets by inhibition entry of the virus on the host cells (Istifli et al., 2020). Similarly, molecular interactions among carvacrol, carvone, and thymol and on borehole spike glycoprotein of SARS‐CoV‐2 showed the binding energy of these phenolic components was found to be in the range of −3.4/−3.7 kcal (Smith & Smith, 2020). The result of a molecular dynamic simulation (MDS) analysis showed that thymol and carvacrol as better inhibitors than camostat as a synthetic inhibitor of TMPRSS2 due to their stable binding 32 with TMPRSS2 in its oxyanion hole (Yadav et al., 2020). TV and its components such as thymol may be useful in COVID‐19‐infected patients by suppressing the destructive effects of the renin–angiotensin system (RAS). Moreover, TV can decrease the pro‐inflammatory mediators and oxidative stress in COVID‐19 infection. The antiviral properties of TV and its bioactive ingredient are shown in Table 1.

**TABLE 1:** Antiviral effects of *T. vulgaris* and its constituent.

Plant/constituent	Type of viruses	Doses	Type of study	Results	References
*T. hyemalis*, *T. vulgaris*, and *T. zygis* extracts	Herpes simplex virus 1 (HSV‐1)	5–15 g/mL	Vero cells	Significantly reduced virus infectivity in the pretreated cells. *T. zygis* was more effective than the other plant extracts	Santoyo et al. (2014)
*T. vulgaris* essential oils	Feline enteric coronavirus (FCoV)	27 and 270 μg/mL	Crandell Reese Feline Kidney (CRFK) cells	Significantly inhibited virus replication. Moreover, reduced virucidal effects by reducing viral titer to 1 h of time contact	Catella et al. (2021)
*T. vulgaris* and *N. cataria* hydrosols	Respiratory syndrome virus (RSV)	2^−2^ to 2^−6^ dilutions	MARC‐145 cells	Inhibited effectively PRRSV infection in vitro	Kaewprom et al. (2017)
				Inhibited viral replication and/or virus release	
Monoterpene including α‐terpinene, *p*‐cymene, γ‐terpinene, and thymol	HSV‐1	High‐purity standards	RC‐37 cells	Reduced viral infectivity and inhibited HSV >80%	Astani et al. (2010)
				Showed high anti‐HSV‐1 effects by directing inactivation with virus particle	
β‐Pinene and limonene	HSV‐1	1–50 (μg/mL)	RC‐37 cells	Reduced viral infectivity by 100% showed anti‐HSV‐1 effects by interactions with free virus particles	Astani and Schnitzler (2014)
Thyme, camomile, and ginger	HSV type 2 (HSV‐2)	0.0001%–0.1%	RC‐37 cells	Reduced plaque formation by more than 90%. Affected HSV‐2 mostly before adsorption may be by interaction with the viral envelopes	Koch et al. (2008)
*T. vulgaris* extracts	Newcastle disease virus (NDV)	10–100 μg/mL	Embryonated eggs	Showed antiviral effects against NDV in embryonated eggs	Rezatofighi et al. (2014)
*T. vulgaris*	COVID‐19	5 g, in 100 mL of water every 8 h	Clinical	Significantly improved cough, dyspnea, anorexia, chest pain, fever, ageusia, and anosmia	Yiagnigni Mfopou et al. (2021)
				Significant decreased blood urea level and neutrophils, while increased lymphocyte count and serum calcium level after 3 days of treatment	
*T. vulgaris* syrup	COVID‐19	5 mL syrup containing 10% thyme essential oil	Clinical	Reduced clinical symptoms such as fever; cough; headache; dyspnea, muscular pain and weakness; anorexia; fatigue; and chest wall pain. Moreover, urea, neutrophil counts; and calcium were significantly decreased, while lymphocyte count was increased	Sardari et al. (2021)

### 4.2 Anti‐inflammatory effects of TV

The quantitatively immunoregulatory properties of TV and its main ingredients on inflammatory/anti‐inflammatory mediators have been reported (Khazdair, Gholamnezhad, et al., 2021a). It was shown that TV aqueous extract (0.01 to 200 μg/mL) significantly reduced the proliferation of mitogen‐induced lymphocytes. The extract also significantly increased expression of CD40 in dendritic cells (DCs) but reduced T‐cell proliferation. The plant extracts did not influence the pattern of mediator secretion (IFN‐γ and IL‐4) by T cells but suppressed proliferation of allogenic T cells (Amirghofran et al., 2012). TV (5, 15, and 25 μg/mL) remarkably reduced the levels of proinflammatory mediators including IL‐6, IL‐1β, and TNF‐α, while the level of an anti‐inflammatory mediator (IL‐10) was increased in stimulated THP‐1 macrophage (Ocaña & Reglero, 2012).

The effects of TV hydroalcoholic extract on lipopolysaccharide (LPS)‐induced lung injury in primary human airway epithelial cell line (H460) significantly reduced protein levels of NF‐κB p52 and NF‐κB p65 transcriptions factors followed by reduction in pro‐inflammatory mediators, IL‐1β and IL‐8, as well as reduced mucus secretion in tracheal epithelial cells and human normal bronchial (Oliviero et al., 2016).

Administration of essential oils of TV, *Cinnamomum verum*, and *Cymbopogon nardus* in LPS‐induced airway inflammation in C57BL/6J mice reduced myeloperoxidase (MPO) activity, inflammatory airway hyperresponsiveness, and certain cellular inflammatory parameters (Csikós et al., 2020).

Treatment of bursal disease of vaccinated and nonvaccinated male broilers with a mixture of crushed TV, *N. sativa* seeds, and *Pimpinella anisum* (*P. anisum*) improved antibody titer in broilers. Furthermore, this mixture treatment improved lymphocytes and monocytes of vaccinated birds (Al‐Beitawi et al., 2010). The supplementation with essential oil of TV (5000 ppm, p.o.) in induced colitis‐inhibited mRNA expression of IL‐1β and reduced macro‐ and microscopic scores of colitis in mice colon (Juhás et al., 2008).

Effects of TV essential oils on sodium hypochlorite induced lung damage in rats showed inhalation of TV (4%) twice a day (30 min) for 4 weeks repeated tissue damage and decreased the histopathological change in the lung tissue. Furthermore, the expression of TNF‐α and oxidative biomarkers were decreased but the total antioxidant status (TAS) was improved compared to control group (Bolatli et al., 2023).

The effects of thymol in LPS‐induced lung inflammation in mice showed administration of thymol (100 mg/kg) inhibited the inflammatory cells influx, protein level in broncho‐alveolar lavage fluid (BALF), TNF‐α and IL‐6 releases, and improved pathological change in lung tissue. Furthermore, thymol inhibited the elevated levels of MPO and MDA and improved reduction in SOD activity (Wan et al., 2018).

Treatment of LPS‐stimulated epithelial cells with thymol (10, 20 and 40 μg/mL) inhibited production of inflammatory cytokines. Thymol also suppressed the gene expressions of inducible nitric oxide synthase (iNOS) and cyclooxygenase‐2 (COX‐2) as dose dependently. In addition, the phosphorylation of c‐Jun N‐terminal kinase (JNK), extracellular signal‐regulated protein kinase (ERK); p38 mitogen‐activated protein kinase (MAPKs), and inhibitor protein of NF‐κB (IκBα) were also inhibited by thymol in stimulate cells (Liang et al., 2014). Treatment of MC/9 cells with thymol (500 to 750 μM) significantly increased gene transcription of IL‐6 and IL‐13 after 3‐hour incubation as dose dependently but did not affect remarkably IL‐6 and IL‐13 in the protein level (Wechsler et al., 2014). Thymol at different concentrations (1.5, 15, and 150 μg/mL) did not remarkably reduce leukocyte migration in response to chemoattractant (fMLP and LTB4), but thymol at dose 150 μg/mL was a potent chemoattractant agent. In addition, oral uses of thymol (100 to 400 mg/kg, p.o.) in carrageenan‐induced acute inflammation in mice remarkably increased leukocyte count, which was comparable to the effects of synergic drugs, celecoxib and indomethacin, 4 hours after injection of carrageenan (Fachini‐Queiroz et al., 2012).

Supplementation therapy of broiler chickens with a mixture of carvacrol and thymol (60 to 200 mg/kg, p.o.) increased IgG anti sheep red blood cells titer, but reduced the ratio of heterophile to lymphocyte in the birds (Hashemipour et al., 2013). Thymol and carvacrol (120 and 240 mg/kg) in dietary supplementation caused upregulation of mRNA expression of toll‐like receptors (TLR)‐2 and IL‐1β in the ileum as well as mucosal secretory IgA in broiler chicken. Moreover, the level of antibodies against NDV was increased in the serum but the mRNA expressions of TLR‐2 and TNF‐α in ileum were inhibited by this supplementation (Du et al., 2016).

Oral administration of thymol at dose of 100 mg/kg or nicotine (2.5 mg/kg, p.o.) alone decreased the level of IL‐1β, TNF‐α, IL‐6, IFN‐γ, and IL‐17 in rheumatoid arthritis induced in animal model in rats. In addition, treatment with thymol and nicotine as combination (half doses with each) more reduced IL‐1β, CRP, IL‐17, and MPO compared to individual thymol and nicotine treatments (Golbahari & Froushani, 2019).

These results indicate that TV and its ingredient, thymol, can alter the pattern of cytokine production and could be used for the treatment of inflammatory, allergic, and immunologic disorders. The anti‐inflammatory and immunoregulatory effects of TV and its main bioactive ingredient are shown in Table 2.

**TABLE 2:** Anti‐inflammatory effects of *T. vulgaris* and its constituent.

Plant/constituent	Doses	Type of study	Effects	References
Aqueous extract	0.01–200 μg/mL	Dendritic cells (DCs)	Reduced lymphocytes	Amirghofran et al. (2012)
			Increased expression of CD40 but reduced T cells proliferation and did not influence the pattern of mediators secretion (IFN‐γ and IL‐4)	
Aqueous extract of *T. vulgaris*	5, 15 and 25 μg/mL	THP‐1‐macrophages	Remarkably reduced the levels IL‐6; IL‐1β; and TNF‐α but increased the level of IL‐10	Ocaña and Reglero (2012)
Hydroalcoholic extract of *T. vulgaris*	–	Human lung cancer cell line (H460)	Significantly reduced protein levels of NF‐κB p65 and NF‐κB p52 transcription factors after decrease in IL‐1‐beta and IL‐8	Oliviero et al. (2016)
			Reduced mucus secretion	
Essential oils of *T. vulgaris*, *Cinnamomum verum*, and *Cymbopogon nardus*	(0.05 mL) Inhalation	Mice	Reduced myeloperoxidase (MPO) activity, inflammatory airway hyper‐responsiveness, and certain cellular inflammatory parameters	Csikós et al. (2020)
*T. vulgaris*, *N. sativa* seeds, and *Pimpinella anisum* (*P. anisum*) crushed	2.0% crushed mixture (1:1:1) as feed additive	Broilers	Improved antibodies titer in broilers. Furthermore, this mixture treatment improved lymphocytes and monocytes of vaccinated birds	Al‐Beitawi et al. (2010)
*T. vulgaris* essential oil	5000 ppm, p.o.	Mice	Inhibited mRNA expression of IL‐1β and reduced macro‐ and microscopic scores of colitis in mouse colon (Juhás et al., 2008)	Juhás et al. (2008)
*T. vulgaris* essential oil	4%	Rats	Repeated tissue damage	Bolatli et al. (2023)
			Decreased the histopathological findings in the lung	
			Decreased expression of TNF‐α and oxidative biomarkers while TAS was improved	
Thymol	100 mg/kg	Mice	Inhibited the protein concentration in BALF	Wan et al. (2018)
			Inhibited inflammatory cell influx, TNF‐α, and IL‐6 release	
			Improved pathological changes in lung tissues	
			Improved oxidative stress biomarkers	
Thymol	10, 20, and 40 μg/mL	mMECs cells	Suppress the gene expression of iNOS and COX‐2	Liang et al. (2014)
			Inhibited ERK and p38 MAPKs	
Thymol	500, 600, and 750 μM	MC/9 cells	Significantly increased gene transcription of IL‐6 and IL‐13 after 3‐hour incubation but did not affect remarkably IL‐6 and IL‐13 in the protein levels	Wechsler et al. (2014)
Thymol	1.5–150 μg/mL	Leukocyte	Thymol 150 μg/mL was a potent chemoattractant agent	Fachini‐Queiroz et al. (2012)
Thymol	100 to 400 mg/kg, p.o.	Mice	Significantly increased leukocyte count	Fachini‐Queiroz et al. (2012)
Mixture of Thymol and carvacrol	60–200 mg/kg, p.o.	Broiler chickens	Increased hypersensitivity response and IgG Decreased the ratio of heterophil to lymphocyte	Hashemipour et al. (2013)
Thymol and carvacrol	120 and 240 mg/kg p.o.	Broiler chickens	Upregulated mRNA expression of IL‐1β, toll‐like receptor (TLR)‐2 in the ileum, and mucosal secretory IgA in broiler chickens	Du et al. (2016)
Thymol or nicotine	100 mg/kg, p.o. or 2.5 mg/kg, p.o.	Rat	Reduced the levels of IL‐1β; TNF‐α; IL‐6; IFN‐γ; and IL‐17 in rheumatoid arthritis in rats. The combination therapy with thymol and nicotine caused more reduction in IL‐1β, CRP, IL‐17, and MPO compared to individual thymol and nicotine treatments	Golbahari and Froushani (2019)

## 5 PHARMACOLOGICAL PROPERTIES OF ZM

*This plant* is traditionally used as antispasmodic, esthetic, antiseptic, antidiarrheal, and analgesic agent (Ghasemi Dehkordi et al., 2002). ZM was also used for dyspepsia, cold, vomiting, headache, as well as premature labor pain in folk remedy. It has been reported that the major constituents of ZM were carvacrol, thymol, and p‐cymene. Moreover, the major constituents of ZM collected from the northeast of Iran are carvacrol, thymol, р cymene, γ‐terpinene, and α‐pinene about 80.19% (Aida et al., 2015). The safety and anti‐antispasmodic, as well as clinical benefit of medicinal plants such as ZM in relieving chronic cough, was also reported (Mortazavi Moghaddam et al., 2020).

### 5.1 Antiviral properties of ZM

The positive properties of ZM in reducing replication of virus in the tissues of H9N2 influenza‐infected broiler chickens were reported. These effects can lead to better performance and clinical symptoms in chickens (Shayeganmehr et al., 2018). Antiviral effect of ZM (800 and 1000 μg/mL) on HSV‐1‐infected vero cells remarkably reduced plaque formation up to 100% (Arabzadeh et al., 2013). The antiviral properties of ZM oil on HSV type 1 in vero cells showed noncytotoxic effect on the cell's viability. ZM oil inhibited virus plaque formation and suggested it could be used as herbal mouthwash (Gavanji et al., 2015). The chemical compositions and antiviral effects of ZM have been reported. The essential oil from ZM contains carvacrol (34.96%), thymol (38%), and paracymene (7.17%). The concentration of 1/10,000 of ZM oil has not inhibited the growth of HSV‐I to Hela cell culture (Khanavi et al., 2010). The antiviral properties of “Oregano oils” and carvacrol against H1N1 virus in canine kidney cells (MDCK) and human lung epithelial cells (A549) showed oils, and carvacrol at concentration (0.01–100 μL/mL) showed antiviral effective (Vimalanathan & Hudson, 2012).

Several studies described bioactivity of carvacrol as ZM ingredient including antibacterial, anti‐inflammatory, antioxidant, antiseptic, antifungal, antiviral, immunomodulatory, and chemopreventive effects (Hashemipour et al., 2013). Carvacrol is able to reduce/inhibit viral disease in human and animal model studies. The antiviral properties of carvacrol (6400 μg/mL) on human respiratory syncytial virus (HRSV), acyclovir‐resistant herpes simplex virus type 1 (ACVRHHV‐1), and human rotavirus (RV) have been reported in vitro (MDBK and HEp‐2 cells) (Pilau et al., 2011).

Treatment of RAW 264.7 cells with carvacrol (0.25% and 0.5%) within 15 min of exposure significantly reduced human noroviruses (NoVs) titers. In addition, with greater duration of exposure to carvacrol, the cell culture infectivity was reduced. Also, the particles of the virus are no longer intact, followed by 30 min expose to carvacrol due to the lack of cells binding in the RNase I protection assay (Gilling et al., 2014).

Exposer to carvacrol (0.25%, 0.5%, and 1%) significantly showed antiviral activity on murine noroviruses (MNV), feline caliciviruses (FCV), and hepatitis A viruses (HAV) in cell cultures by reduction in infectivity and viral contamination, and completely inactivate the two norovirus surrogates (Sánchez et al., 2015). Exposed with carvacrol (100 μM) on HSV‐1 **infect vero cells** decreased herpes up to 70%. Carvacrol has no effect on replication, attachment, and penetration of virus (Kamalabadi et al., 2018).

Treatment of infected BSC‐1 cells by HSV‐2 with carvacrol (1, 0.5, 0.25, 0.125, and 0.0625 mmol/L) inhibited the expressions of transcription genes (ICP27, VP16, ICP4, and UL30) and protein levels of cytokines such as RIP3, MLKL, and TNF‐α due to viral replication as dose dependently (Wang et al., 2020). It has been reported that carvacrol could serve as an inhibitor in regulating the main protease (M^pro^) of COVID‐19 functions and controlling viral replications (Kumar et al., 2021). Antiviral properties of ZM and its bioactive ingredient are shown in Table 3.

**TABLE 3:** Antiviral effects of *Z. multiflora* and its main constituents.

Plant/constituent	Type of viruses	Doses	Type of study	Results	References
*Z. multiflora*	HSV‐1	800 and 1000 μg/mL	Vero cells	Remarkably reduced plaque formation up to 100%	Arabzadeh et al. (2013)
*Z. multiflora* oil	HSV‐1	0.02%–0.4%	Vero cells	Showed noncytotoxic effect on the cell's viability. *Z. multiflora* oil inhibited virus plaque formation and suggested it could be used as herbal mouthwash	Gavanji et al. (2015)
*Z. multiflora* oil	HSV‐1	1/10,000	Hela cells	The concentration of 1/10000 of *Z. multiflora* oil has not inhibited the growth of HSV‐I to cell culture	Khanavi et al. (2010)
Oregano oils and carvacrol	H1N1	0.01–100 μL/mL	MDCK and A549	Showed potent antiviral effectiveness	Vimalanathan and Hudson (2012)
Carvacrol	ACVRHHV‐1), HRSV, and RV	6400 μg/mL	MDBK and HEp‐2 cells	Showed antiviral activity by inhibiting the viruses by virus infection and replication	Pilau et al. (2011))
Carvacrol	NoVs	0.25% and 0.5%	RAW 264.7 cells	Treatment with carvacrol after 15 min of exposure reduced norovirus titers. In addition, cell culture infectivity was reduced. Virus particles are no longer intact following 30 min exposure to carvacrol	Gilling et al. (2014)
Carvacrol	FCV, MNV, and HAV	0.25, 0.5, and 1%	RAW 264.7, CRFK, and FRhK‐4 cells	Significantly showed activity in cell cultures by reduction in infectivity and viral contamination	Sánchez et al. (2015)
Carvacrol	HSV‐1	100 μM	**Vero cells**	Decreased herpes up to 70%. Carvacrol has no effect on replication, attachment, and penetration of virus	Kamalabadi et al. (2018)
Carvacrol	HSV‐2	1, 0.5, 0.25, 0.125, and 0.0625 mmol/L	BSC‐1 cells	Inhibited the expressions of transcription genes and protein levels of cytokines due to viral replication	Wang et al. (2020)

### 5.2 Anti‐inflammatory properties of ZM

The aqueous extract of ZM (0.1–100 μg/mL) reduced the proliferation of lymphocytes without affecting remarkable cytotoxic effect on DCs. The plant extract showed no effect on the secretions of IFN‐γ and IL‐4 mediators, while increasing the expression of CD40 in DCs (Amirghofran et al., 2012). ZM extract (200–800 μg/mL) in splenocyte cell culture from sensitized mice significantly increases expressions of IFN‐γ and FOXP3 but decreases gene expressions of TGF‐β and IL‐17 (Kianmehr et al., 2017). ZM hydroethanolic extract (50) reduced expression of IL‐4 at gene and protein but the higher dose of extract (200 μg/mL) increased remarkably the genes expression of IFN‐γ in PHA‐stimulated peripheral blood mononuclear cell (PBMC) (Boskabady et al., 2013).

Treatment of OVA‐sensitized guinea pigs with ZM extracts (0.2–0.8 mg/mL) enhanced the serum level of IFN‐γ, whereas decreased the level of IL‐4 (Boskabady et al., 2013). Subcutaneous injection of ZM essences (200 mg/kg, p.o. and s.c.) stimulated the immune response against *Candida albicans* antigens and Con‐A mitogen in the animals. Moreover, treatment with ZM (200 mg/kg, s.c.) at subcutaneous administration led to more stimulatory effect on the immune system in comparison to oral administrations (Khosravi et al., 2007).

ZM extract (0.2–0.8 mg/mL) also significantly improved the level of eosinophil peroxidase (EPO) and IgE in the serum of guinea pig sensitized by OVA. Also, the extract (0.8 mg/mL, p.o.) was higher affected on most parameters than dexamethasone (Boskabady, Tabatabaee, et al., 2014). Treatment of cigarette‐induced COPD in animal models with extracts of ZM (0.4–1.6 mg/mL, p.o.) significantly reduced IL‐8 levels and eosinophil counts compared to untreated COPD group (Boskabady & Gholami Mhtaj, 2014).

The effects of ZM hydroalcoholic extracts (200 and 800 mg/kg/day) on inhaled paraquat (PQ) induced lung inflammation significantly reduced levels of IL‐6, malondialdehyde (MDA), and nitrite (NO_2_), but enhanced the level of IFN‐γ compared to control animals (Amin et al., 2020).

Administration of hydroalcoholic extracts of ZM (200 and 800 mg/kg/day) on PQ‐induced memory and lung injury in rats significantly reduced total and different WBC counts, IL‐10, NO_2_, INF‐γ, and MDA levels, while enhancing the catalase (CAT) and superoxide dismutase (SOD) activities as well as thiol levels (Heydari et al., 2021).

The results of a randomized clinical study showed administration of ZM extract (5 and 10 mg/kg, p.o.) in sulfur mustard exposer patient (long‐term) lung injury for 2 months remarkably reduced the levels of inflammatory mediators, IL‐6, IL‐2, and IL‐8, but it increases the levels of mediators, IL‐10 and IFN‐γ, compared to the placebo‐treated patient (Khazdair et al., 2019).

In the other clinical trial treatment of asthmatic and COPD patients with 5 and 10 mg/kg concentrations of ZM extract for 2 months, remarkably increased pulmonary function test values but reduced respiratory symptoms in treated groups. The laboratory results showed total WBC, monocyte and eosinophils, hemoglobin, and hematocrit were reduced in treated asthmatic and COPD patients. Furthermore, the levels of inflammatory mediators including TNF‐α, hs‐CRP, and IL‐8 declined in the treatment groups (Alavinezhad et al., 2022; Ghorani et al., 2022).

Exposed of carvacrol (0.3 to 90 μg/mL) decreased leukocyte chemotaxis as dose dependently, which had been induced by chemoattractants, namely fMLP and LTB_4_ (Fachini‐Queiroz et al., 2012). Carvacrol (75–300 μg/mL) significantly enhanced the gene expression of FOXP3 and IFN‐γ but significantly reduced gene expression of IL‐17, TGF‐β, and IL‐4 in splenocytes obtained from sensitized rats. Carvacrol at higher doses also significantly increases IFN‐γ/IL‐4 ratio compared to control group (Kianmehr et al., 2016).

Treatment of splenocyte supernatant obtained from OVA‐immunized mice with carvacrol (80 mg/kg, i.p.) decreased the mRNA expression of, IL‐5, IL‐17A, IL‐4, and IL‐23α, as well as the expression of Th1 cytokines (IL‐2 and IFNγ). In addition, the gene expression of IL‐10 and TGFβ also remarkably increased in the carvacrol‐treated cells compared to the untreated cells (Gholijani & Amirghofran, 2016). Treatment of OVA ‐sensetized guinea pigs with carvacrol (40 to 160 μg/mL) cased to increases in the level of IFN‐γ, but reduction in the level of IL‐4 and endothelin in the serum of animals (Jalali et al., 2013).

Administration of carvacrol (1000–5000 mg/kg) in rainbow fish showed no particular effect on the hematological parameter. However, administration of carvacrol at higher doses significantly increased serum lysozyme activity after 30 days. Also, carvacrol significantly increased the myeloperoxidase activity on day 30. Carvacrol (5000 mg/kg) significantly enhanced serum of total protein, myeloperoxidase activity, globulin, and triglyceride levels in the treated compared to the control group after day 60 (Yilmaz & Ergün, 2015). Carvacrol (40–160 μg/mL) significantly improved the levels of IgE and EPO in the serum of OVA‐induced asthma in sensitized guinea pigs compared to control group (Boskabady, Tabatabaee, et al., 2014).

Treatment of LPS‐induced inflammation in rats with carvacrol (25–100 mg/kg, i.p.) remarkably reduces IL‐6 in the brain (Hakimi et al., 2020). Carvacrol (15 mg/kg, p.o.) for 17 days reduces the value of absolute eosinophil counts (AEC), immunoglobulin E (IgE), IL‐4, TNF‐α, IL‐5, IFN‐γ, IL‐13, iNOS, and MDA levels in the serum of OVA‐induced asthma in rats. Moreover, it significantly improved histopathological changes and increased the value of SOD and GSH compared to control group (Ezz‐Eldin et al., 2020). The effects of carvacrol on cadmium (Cd)‐induced lung toxicity in rats showed that carvacrol (25 or 50 mg/kg) oral intake 30 min after Cd administration exhibited antioxidant properties and suppressed pro‐inflammatory cytokines by lowering *NF*‐*κB* and *p38* mitogen‐activated protein kinase (p38 MAPK) signaling. It also decreases the levels of Bax, cytochrome c, and caspase‐3 that are increased by Cd. In addition, carvacrol reduces levels of 8‐hydroxy‐2′‐deoxyguanosin (8‐OHdG), matrix metallopeptidase (MMP)‐2 and 9, which increased with Cd (Yesildag et al., 2022).

The pharmacological effect of carvacrol on several clinical trials was investigated. Carvacrol reduced respiratory symptoms; serum levels of IL‐2, IL‐4, and IL‐6; vascular endothelial growth factor (VEGF); and epidermal growth factor (EGF) remarkably, while improved the levels of IL‐10 and IFN‐γ in the treatment of SM‐exposed patients compared to control group (Khazdair & Boskabady, 2019a, 2019b). Treatment of asthmatic patients with carvacrol (1.2 mg/kg, p.o.) for 2 months improved oxidative stress markers and significantly increased pulmonary function test values after treatment. It also remarkably enhanced the levels of IL‐10 and IFN‐γ in the serum, while remarkably decreasing the level of IL‐4 (Ghorani et al., 2021). The results of the other clinical study showed carvacrol (1.2 mg/kg, p.o.) improved pulmonary function test values, respiratory symptoms, and hematological indices. It also significantly reduces total and differential WBC and serum levels of hs‐CRP (Alavinezhad et al., 2018).

The results of the above studies indicated that ZM and its main ingredient, carvacrol, reduced pro‐inflammatory cytokines production and suggested their use for treatment of inflammatory and allergic disorders. The anti‐inflammatory effects of *Z. multiflora* and its main bioactive ingredient are shown in Table 4.

**TABLE 4:** Anti‐inflammatory effects of *Z. multiflora* and its main component, carvacrol.

Plant/constituent	Doses	Type of study	Results	References
Aqueous extract	0.1, 1, 50, and 100 μg/mL	DCs	Reduced the proliferation of lymphocytes without causing remarkable cytotoxic effects	Amirghofran et al. (2012)
			Increased the expression of CD40 in DCs	
Hydroethanolic extract	200–800 μg/mL	Splenocytes from sensitized mice	Increased gene expressions of IFN‐γ and FOXP3	Kianmehr et al. (2017)
			Decreased gene expressions of TGF‐β and IL‐17	
Hydroethanolic extract	50–200 μg/mL	PBMCs	Reduced the gene and protein expression of IL‐4	Boskabady et al. (2013)
			Enhanced the gene expression of IFN‐γ	
Essences	200 mg/kg, s.c.	Rabbit	Subcutaneous administration led to more stimulatory effects on the immune system	Khosravi et al. (2007)
Hydroethanolic extract	0.2–0.8 mg/mL, p.o.	Guinea pig model of asthma	Improved the levels of IgE and EPO in guinea pigs sensitized by ovalbumin	Boskabady, Tabatabaee, et al. (2014)
Aqueous extract	0.4–1.6 mg/mL, p.o.	Guinea pig model of COPD	Significantly reduced IL‐8 levels and eosinophil counts	Boskabady and Gholami Mhtaj (2014)
Hydroethanolic extract	200 and 800 mg/kg/day, p.o.	PQ‐induced lung injury in rat	Reduced levels of IL‐6, MDA, and NO_2_	Amin et al. (2020)
			Enhanced the level of IFN‐γ	
Hydroethanolic extract	200 and 800 mg/kg/day, p.o.	PQ‐induced memory and lung damage in rat	Significantly reduced total and different WBC count; IL‐10; INF‐γ; NO_2_; and MDA levels	Heydari et al. (2021)
			Increased thiol levels and CAT and SOD activities	
Syrup	5 and 10 mg/kg/day, p.o	Clinical, sulfur mustard‐induced lung injury	Significantly reduced the levels of IL‐2, IL‐6, and IL‐8	Khazdair et al. (2019)
			Enhanced the levels of IL‐10 and IFN‐γ	
Syrup	5 and 10 mg/kg/day, p.o	Clinical, asthmatic patients	Significantly increased pulmonary function test values	Alavinezhad et al. (2022)
			Reduced respiratory symptom	
			Total WBC, monocyte and eosinophils, hemoglobin, and hematocrit were decreased	
Syrup	5 and 10 mg/kg/day, p.o	Clinical, COPD patients	Pulmonary function test values were increased	Ghorani et al. (2022)
			Decreased TNF‐α and IL‐8 at serum	
Carvacrol	0.3–90 μg/mL	Leukocyte	Reduced leukocyte chemotaxis induced by fMLP and LTB_4_	Fachini‐Queiroz et al. (2012)
	75, 150, and 300 μg/mL	Splenocytes from sensitized rats	Enhanced the gene expression of FOXP3 and IFN‐γ	Kianmehr et al. (2016)
			Reduced IL‐4, TGF‐β, and IL‐17 genes expression	
			Increased IFN‐γ/IL‐4 ratio	
	80 mg/kg, i.p. to OVA‐sensitized mice	Splenocytes	Reduced the mRNA expression of IL‐4, IL‐17A, IL‐5, and IL‐23α	Gholijani and Amirghofran (2016)
			Increased the gene expression of TGFβ and IL‐10	
	40 to 160 μg/mL	Guinea pigs	Increased the levels of IFN‐γ	Jalali et al. (2013)
			Reduced levels of IL‐4 and endothelin	
	1000, 3000, or 5000 mg/kg, p.o.	Rainbow trout fish	Increased serum lysozyme activity, myeloperoxidase activity, myeloperoxidase activity, serum total protein, globulin, and triglyceride levels	Yilmaz and Ergün (2015)
	25–100 mg/kg, i.p.	Rat	Remarkably reduced IL‐6	Hakimi et al. (2020)
	15 mg/kg, p.o.	Rat	Reduced the values of AEC, IgE, IL‐4, IL‐13, TNF‐α, IL‐5, IFN‐γ, iNOS, and MDA	Ezz‐Eldin et al. (2020)
			Improved histopathological changes and increased the values of SOD and GSH	
	25 or 50 mg/kg	Rat	Exhibited antioxidant properties and suppressed *NF*‐*κB* and p38 MAPK signaling	Yesildag et al. (2022)
			Decreased the levels of Bax, caspase‐3, and cytochrome c	
			Reduce levels of 8‐OHdG, MMP‐2, and 9	
	1.2 mg/kg, p.o.	Clinical, sulfur mustard‐induced lung injury	Decreased respiratory symptoms, and serum levels of VEGF, EGF, IL‐6, IL‐2, IL‐4, and IL‐8	Khazdair and Boskabady (2019a, 2019b)
			Enhanced the levels of IL‐10 and IFN‐γ	
	1.2 mg/kg, p.o.	Clinical, asthmatic patients	Improved oxidative stress markers	Ghorani et al. (2021)
			Increased pulmonary function test values after treatment	
			Enhanced the levels of IL‐10 and IFN‐γ in serum	
			Significantly decreased the concentration of IL‐4 in serum	
	1.2 mg/kg, p.o.	Clinical, COPD patients	Improved respiratory symptoms and PFT as well as hematological indices	Alavinezhad et al. (2018)
			Significantly reduced total and differential WBC and serum levels of hs‐CRP	

## 6 ANTIOXIDANT EFFECTS OF HERBS AND THEIR CONSTITUENTS

Oral treatment of aflatoxin‐induced oxidative stress in rats with essential oil *of* TV (5 and 7.5 mg/kg) significantly decreased AST, ALP, ALT, Cho, TriG, total lipids, and creatinine in the serum, but significantly increased total antioxidant capacity (TAC) level in the liver tissue. The extract also significantly decreased MDA level in liver and kidney of treated animals compared to control group (El‐Nekeety et al., 2011). Effects of TV extract (500 mg/kg/day, p.o.) against lead (Pb)‐intoxicated rats remarkably improved all changes in Pb‐induced toxicity, attenuated the levels of IL‐6, IL‐1β, and TNF‐α, while enhancing the levels of IFN‐γ and IL‐10 in the serum. TV extract also increased SOD, CAT, GSH, and glutathione peroxidase (GPx) but reduced the level of MDA (El‐Boshy et al., 2019). Administration of thyme water extract (100 mL/L) in their drinking water increased total oxidant status and total antioxidant response, while lowered the rate of DNA damage in Japanese quails (Sengül et al., 2008). ZM extract has high amount of phenolic compounds with potent antioxidant activities (Alizadeh & Shaabani, 2014). The hydroalcoholic extracts of ZM showed strong radical scavenging activity (~71%) compared to vitamin C (~48%) as a standard antioxidant agent. Additionally, ZM can inhibit the formation of peroxidation products by ~95% (Fatemi et al., 2012).

The essential oils of ZM and its methanolic extract showed antioxidant properties using two standard methods for inhibition of free radicals such as ammonium thiocyanate systems and DPPH (Moshafi et al., 2007; Sharififar et al., 2007). Furthermore, the essential oils from five ecotypes of ZM showed the potential antioxidant capacity that was assessed by DPPH methods. These results indicate that all essential oils from the plant have remarkable antioxidant properties (Saei‐Dehkordi et al., 2010). Therefore, the potent antioxidant activity of the plant was approved by different methods.

Treatment of human monocyte culture (HMC) in the presence of glucose with ZM showed antioxidant effects including: reduced H_2_O_2_ and nitric oxide (NO) production, inhibited NO synthase (NOS) and NADH oxidase (NOX) activities, and prevented nitrosative stress and lipid peroxidation in these cells (Kavoosi & Teixeira da Silva, 2012).

Treatment of cyclophosphamide‐induced oxidative stress by extract of ZM (50–200 mg/kg, b.w.) in mouse bone marrow cells showed antioxidant activity on DPPH and lipid peroxidation (Hosseinimehr et al., 2010). The possible beneficial effect of ZM extract (50–200 μL/kg) on cognitive function in Alzheimer's disease rat model showed that ZM treatment reduces total NO and nitrite levels in the serum of animals (Majlessi et al., 2012).

The ethanol extract of ZM on the hyperglycemia mice induced by bisphenol A (BPA) reduced the level of MDA content but increased GSH in the serum of animals. ZM (50–400 mg/kg, i.p.) caused pulmonoprotective effect by inhibition of MDA production and reduction in glutathione content, and SOD and CAT activities induced by cyclophosphamide (CP) in the lung (Habibi et al., 2014).

The potent antioxidant effects of carvacrol on MDA and NO scavenging activity (Karimian et al., 2012), and antioxidant effects for thymol by reduction in H_2_O_2_ and NO production and NO synthase activities in glucose‐stimulated HMC were shown (Kavoosi & Teixeira da Silva, 2012). Treatment of LPS‐stimulated murine macrophages with thymol and carvacrol remarkably reduced H_2_O_2_ and NO production as well as NADH oxidase activity and NO synthase (Kavoosi et al., 2012). Administration of carvacrol at a dose of 73 mg/kg on methotrexate (MTX)‐induced inflammation and oxidative stress significantly attenuated MDA, total oxidant status (TOS), and IL‐1 𝛽 and TNF‐α levels, while increasing the TAS compared to the MTX group (Celik et al., 2013). Treatment of animal model of Parkinson's disease with carvacrol (10 mg/kg) reduced the level of nitrite and MDA as well as increased antioxidant enzyme compared to control animals (Hassanshahi et al., 2014).

Treatment of radiation‐induced cytotoxicity in the cells (V79) with thymol significantly increased glutathione, CAT, and SOD enzyme levels, but reduced the level of MDA in V79 cells. Additionally, thymol could antagonize toxicity by normalizing the antioxidant levels (Archana et al., 2009). These results showed that the monoterpenes, thymol, and carvacrol contribute to antioxidant properties. The antioxidant effects of medicinal herbs and their ingredients are summarized in Table 5.

**TABLE 5:** Antioxidant effects of *T. vulgaris*, *Z. multiflora*, and their constituents.

Plant Extract/constituents	Doses	Model of study	Results	References
*T. vulgaris* oil	5 and 7.5 mg/kg b.w.	Aflatoxin‐induced oxidative stress in rats	Decreased serum AST; ALT; ALP; TriG; Cho; total lipids; and creatinine	El‐Nekeety et al. (2011)
			Increased TAC level	
			Decreased MDA level in liver and kidney	
*T. vulgaris*	500 mg/kg/day, p.o.	Pb‐intoxicated rats	Improved IL‐6; IL‐1β; and TNF‐α at serum levels	El‐Boshy et al. (2019)
			Increased IL‐10 and IFN‐γ	
			Increased GPx; GSH; CAT; and SOD	
			Reduced the level of MDA	
Thyme water extract	100 mL/L	Japanese quails	Increased total oxidant status and total antioxidant response, while lowered rate of DNA damage in Japanese quails	Sengül et al. (2008)
*Z. multiflora*	50, 100, and 200 mg/kg b.w.	Mouse bone marrow cells	Showed antioxidant activity on DPPH and lipid peroxidation.	Hosseinimehr et al. (2010)
*Z. multiflora*	50, 100, or 200 μL/kg	Rat model of Alzheimer's disease	Reduced total NO and nitrite in serum	Majlessi et al. (2012)
*Z. multiflora*	0.2, 0.4, and 0.8 mg/mL	Guinea pig model of asthma	Reduced serum levels of total NO and nitrite	Boskabady, Jalali, et al. (2014)
*Z. multiflora*	400, 600, and 900 p.p.m	Mice model of IBD	Increased activity of MPO and lipid peroxidation products	Nakhai et al. (2007)
*Z. multiflora*	0.4, 0.8, and 1.6 mg/mL	Guinea pig model of COPD	Increased thiol groups in BALF	Boskabady and Mahtaj (2015)
Thymol and carvacrol	1, 10, and 100 ng/mL	Human monocytes stimulated by glucose	Reduced production of NO and H_2_O_2_	Kavoosi and Teixeira da Silva (2012)
			Reduced NADH oxidase and NO synthase activity	
Thymol and carvacrol	1, 10, and 100 ng/mL	LPS‐stimulated murine macrophages	Reduced NO, H_2_O_2_, NO synthase, and NADH oxidase activity	Kavoosi et al. (2012)
Thymol	5, 10, 25, 50, 75, and 100 g/mL	Hamster lung fibroblast cells	Increased intracellular level of GSH, CAT, and SOD enzyme	Archana et al. (2009)
Carvacrol	73 mg/kg, i.p.	MTX‐induced oxidative stress	Reduced MDA, TOS, TNF‐α, and IL‐1 𝛽 levels	Celik et al. (2013)
			Increased TAS compared to the MTX group	
Carvacrol	10 mg/kg	6‐Hydroxydopamine‐induced Parkinson's disease	Decreased the rotation numbers and MDA	Hassanshahi et al. (2014)
			Enhanced the nitrite level	

Abbreviations: ALP, alkaline phosphatase; ALT, alanine transaminase; AST, aspartate aminotransferase; CAT, catalase; *DPPH*, 2,2‐diphenylpicrylhydrazyl; GGT, gamma‐glutamyl transferase; GPx, glutathione peroxidase; GSH, glutathione; MDA, malondialdehyde; NO_2_, nitrite; SOD, superoxide dismutase; TAC, total antioxidant capacity; TOS, total oxidant status.

## 7 CONCLUSION

This review study descriptively highlights the possible therapeutic activities of some Lamiaceae family plants and their major bioactive components with their possible mechanisms of action on coronavirus‐induced lung inflammation. According to our literature review, TV and ZM with their major ingredients have various properties such as antiviral effects in different studies, stimulation and modulation of immune responses, improvement in IgE serum levels and eosinophil counts, reduction in the levels of pro‐inflammatory mediators (IL‐1β, IL‐17, IL‐6, IL‐4, and TGF‐β), as well as enhancement of anti‐inflammatory mediators (IFN‐γ and FOXP3). These medicinal herbs and their constituents also enhance antioxidant capacity. The plant constituents could inhibit the main protease (M^pro^) of coronavirus functions and control viral replications and inhibition, as well as interaction with the cellular proteases TMPRSS2 could inhibit the entry of the virus on the host cells. In addition, *these medicinal plants* improved pulmonary function tests and respiratory symptoms in obstructive lung disease. In this regard, ARDS with cytokine storm of pro‐inflammatory cytokines is the main death cause of coronavirus and *these plants* with anti‐inflammatory and immunomodulatory effects as well as protective effects on obstructive diseases, it may be useful for treatment of coronavirus‐induced lung inflammation. However, more clinical studies are required to support drug effectiveness.

## AUTHOR CONTRIBUTIONS

**Majid Kianmehr:** Conceptualization (equal); data curation (equal); methodology (lead); writing – original draft (equal). **Mohammad Reza Khazdair:** Conceptualization (lead); data curation (equal); methodology (lead); writing – original draft (lead); writing – review and editing (lead). **Abbasali Abbasnezhad:** Conceptualization (equal); methodology (equal); writing – original draft (equal). **Muhammad Akram:** Conceptualization (equal); writing – original draft (equal); writing – review and editing (equal).

## FUNDING INFORMATION

The author(s) declare not receiving external funds for the research and/or publication of this article.

## CONFLICT OF INTEREST STATEMENT

The authors have no conflicts of interest to declare for this study.

## ETHICS STATEMENT

The current study is a review article and did not involve direct experimentation on any animals and humans and no ethical approval was required.

## Data Availability

All data that support the findings of this study are included in this review article.
